# Identification of single nucleotide polymorphism markers associated with bacterial cold water disease resistance and spleen size in rainbow trout

**DOI:** 10.3389/fgene.2015.00298

**Published:** 2015-09-24

**Authors:** Sixin Liu, Roger L. Vallejo, Yniv Palti, Guangtu Gao, David P. Marancik, Alvaro G. Hernandez, Gregory D. Wiens

**Affiliations:** ^1^National Center for Cool and Cold Water Aquaculture, Agricultural Research Service, United States Department of AgricultureKearneysville, WV, USA; ^2^Roy J. Carver Biotechnology Center, University of Illinois at Urbana-ChampaignUrbana, IL, USA

**Keywords:** rainbow trout, *Flavobacterium psychrophilum*, bacterial cold water disease, spleen size, SNP, QTL

## Abstract

Bacterial cold water disease (BCWD) is one of the frequent causes of elevated mortality in salmonid aquaculture. Previously, we identified and validated microsatellites on chromosome Omy19 associated with QTL (quantitative trait loci) for BCWD resistance and spleen size in rainbow trout. Recently, SNPs (single nucleotide polymorphism) have become the markers of choice for genetic analyses in rainbow trout as they are highly abundant, cost-effective and are amenable for high throughput genotyping. The objective of this study was to identify SNP markers associated with BCWD resistance and spleen size using both genome-wide association studies (GWAS) and linkage-based QTL mapping approaches. A total of 298 offspring from the two half-sib families used in our previous study to validate the significant BCWD QTL on chromosome Omy19 were genotyped with RAD-seq (restriction-site-associated DNA sequencing), and 7,849 informative SNPs were identified. Based on GWAS, 18 SNPs associated with BCWD resistance and 20 SNPs associated with spleen size were identified. Linkage-based QTL mapping revealed three significant QTL for BCWD resistance. In addition to the previously validated dam-derived QTL on chromosome Omy19, two significant BCWD QTL derived from the sires were identified on chromosomes Omy8 and Omy25, respectively. A sire-derived significant QTL for spleen size on chromosome Omy2 was detected. The SNP markers reported in this study will facilitate fine mapping to identify positional candidate genes for BCWD resistance in rainbow trout.

## Introduction

Bacterial cold water disease (BCWD) is one of the frequent causes of elevated mortality in salmonid aquaculture (Nematollahi et al., 2003; Starliper, 2011; Loch and Faisal, 2015). The etiological agent of BCWD is a Gram-negative bacterium, *Flavobacterium psychrophilum*, which also causes rainbow trout fry syndrome in small fish. Currently, there is no licensed commercial vaccine for BCWD. Use of the limited antibiotics available in food fish can increase the production costs and is a concern for emergence of antibiotic-resistant pathogens. Fortunately, host resistance can be improved through selective breeding (Leeds et al., 2010), and a fall-spawning line with improved BCWD resistance evaluated in both laboratory and on-farm tests has been developed (Wiens et al., 2013a). Currently, there is interest in improving BCWD resistance of other populations of different origin and spawn times. Conventional family based selection for BCWD resistance relies on indirect phenotype evaluation of siblings, which does not take advantage of the genetic variation within families. The development of molecular markers associated with BCWD resistance would facilitate marker-assisted selection for BCWD resistance and enable direct selection of breeding candidates.

As the first step to implement marker assistant selection and eventual positional cloning of genes for BCWD resistance, we have conducted several studies to map QTL (quantitative trait loci) for BCWD resistance in rainbow trout. Because rainbow trout mature after 2 years of growth, there are two year-class breeding populations: even-year population and odd-year population. Previously, we used microsatellites to identify QTL for BCWD resistance in either even or odd-year populations of rainbow trout. Nine major QTL on seven chromosomes were identified in six odd-year mapping families (Vallejo et al., 2014a). In an even year mapping family, 2008132, a major QTL for BCWD resistance on chromosome Omy19 was identified (Wiens et al., 2013b). Interestingly, a major QTL for spleen index on chromosome Omy19 was also identified in the same mapping family, which was consistent with the association between BCWD resistance and spleen size in rainbow trout reported in our previous study (Hadidi et al., 2008). Both QTL for BCWD resistance and spleen size on chromosome Omy19 were validated in a subsequent generation (Vallejo et al., 2014b), and the QTL mapping results suggest that two separate QTL on chromosome Omy19 control BCWD resistance and spleen size, respectively.

The microsatellites available in rainbow trout are limited, and microsatellite genotyping for whole-genome scans is no longer cost effective while the cost of SNP (single nucleotide polymorphism) genotyping is decreasing. SNPs have become the markers of choice as they are highly abundant and are amenable for high throughput genotyping. RAD-seq (restriction-site-associated DNA sequencing) targets sequences surrounding the restriction enzyme cut site (Miller et al., 2007; Baird et al., 2008), which has rapidly become a cost-effective method of SNP genotyping in aquaculture species as it does not require a prior marker discovery or a reference genome sequence. Recently, Campbell et al. (2014) reported SNP markers associated BCWD resistance in rainbow trout using RAD-seq. Similarly, we have also used RAD-seq to identify SNPs associated with QTL for stress response (Liu et al., 2015) and for BCWD resistance in odd-year mapping families (Palti et al., 2015b). In this study, offspring of two dam half-sib even-year mapping families used previously for microsatellites-based validation of the QTL for BCWD resistance on chromosome Omy19 (Vallejo et al., 2014b) were genotyped using RAD-seq. The objective of this study was to identify SNPs associated with BCWD resistance and spleen size using both genome-wide association studies (GWAS) and linkage-based QTL mapping approaches.

## Materials and Methods

### Ethic Statement

The experiment was conducted in accordance of protocol #47, approved by the Institutional Animal Care and Use Committee, National Center for Cool and Cold Water Aquaculture, Agricultural Research Service, United States Department of Agriculture.

### Mapping Families and Phenotyping

Two dam half-sib mapping families 2012473 and 2012474 were used in this study. Family 2012473 was made from the cross between 2008132002 and 2009044043, and family 2012474 was made from the cross between 2008132002 and 2009044044. The 4-year-old female fish 2008132002 was derived from the mapping family 2008132 used previously for BCWD QTL mapping in even–year class fish (Wiens et al., 2013b). Both 3-year-old male fish 2009044043 and 2009044044 were derived from the mapping family 2009044 used previously for BCWD QTL mapping in odd-year class fish (Vallejo et al., 2014a). These two mapping families were chosen for this study because the shared dam 2008132002 is segregating for the BCWD QTL but not for the spleen index QTL on chromosome Omy19 (Vallejo et al., 2014b). In addition, the sire of family 2012474 (fish 2009044044) is segregating for the QTL for spleen index on chromosome Omy19 (Vallejo et al., 2014b).

The disease challenge and collection of phenotype data were described in our previous publication (Vallejo et al., 2014b). Briefly, 100 fish from each mapping family were challenged with BCWD strain CSF259-93 by intraperitoneal injection at an average of 62 days post-hatch. Mortalities were collected each day for 21 days after intraperitoneal injection. Survival days (DAYS), the number of days to death post challenge, were recorded and survivors at the end of the experiment were assigned a value of 22 days. Each individual fish also had a record of survival status (STATUS). The binary trait STATUS had two classes: fish died during the 21 days period post challenge were assigned a value of 1, and fish alive on day 22 post-challenge were assigned a value of 2. Phenotypes DAYS and STATUS were used for GWAS and QTL mapping for BCWD resistance. Both body weight and spleen weight were recorded for 98 naïve 182-day-old fish from the family 2012474. Spleen index for each fish was calculated using the formula 2.1 × [spleen weight (mg)/body weight (g)] (Vallejo et al., 2014b).

### RAD Genotyping

Genomic DNA of three mapping parents and 298 offspring of mapping families 2012473 and 2012474 was digested with restriction enzyme SbfI, and RAD sequencing libraries were prepared as described in Palti et al. (2014). Each RAD library containing 30 to 32 indexed samples with a unique six-nucleotide barcode was sequenced (single end 100 bp read) on a single lane of HiSeq 2000. Raw sequences were submitted to the short read archive of GenBank under accession number SRP062268. After trimming the six-base barcode at the 5′ end and the last five bases at the 3′ end of each sequence read, we filtered out reads with a cumulative sequencing error probability of more than 20% in the 89 bp read (i.e., call probability better than 99.5% per base for the sequences that passed filtering). The remaining trimmed reads were processed to identify SNPs using Novoalign and Perl scripts (Palti et al., 2014). SNP loci and samples with more than 30% missing data were removed from the final genotype data (passing call rate >70%). Also, Chi-square goodness-of-fit tests were used to check the genotype segregation ratio (1:1 or 1:2:1) of each SNP, and SNPs with Bonferroni-corrected segregation distortions (*P* < 1e-5) were dropped from the final genotype dataset.

### Construction of SNP Linkage Maps

The SNP genotypes of these two mapping families 2012473 and 2012474 were combined to construct linkage maps using software MULTIMAP (Matise et al., 1994). Based on two-point analysis, SNPs were assigned to 29 linkage groups with parameters of LOD 10 and recombination fractions less than 0.2. The SNP sequences were mapped to the reference genome sequence of rainbow trout (Berthelot et al., 2014). Based on the chromosomal assignments of majority SNPs in each linkage group, the 29 linkage groups were assigned to chromosomes of rainbow trout. We used sire-specific and dam-specific SNPs in each linkage group to construct male and female genetic maps, respectively. Due to the large number of SNPs in each linkage groups, it was challenging to build linkage maps using directly the default threshold of LOD 3. Thus, the initial linkage map was constructed with a threshold of LOD 10. For each linkage group where the linkage map was smaller than 150 cM, more SNPs were added to the linkage map using a threshold of LOD 5. If the linkage group map length was still smaller than 150 cM with the threshold of LOD 5, the threshold was lowered to LOD 3 to add more SNPs to the map.

### Genome Wide Association Analysis

Single nucleotide polymorphism markers that were assigned to chromosomes through two-point linkage analysis and had over 70% genotype call rates were used for GWAS using the Grammar-Gamma method (Svishcheva et al., 2012) implemented in the R package GenABEL (Aulchenko et al., 2007). This method runs a single-point association test accounting for family relatedness and includes two analysis steps. First, we performed polygenic analysis modeling family relationships; the genomic kinship matrix estimated from SNPs was included in the model of association analysis. Second, we performed association analysis using the residuals from step 1 analysis. Thus, corrected SNPs effect were used in the association tests. We used the threshold *P* < 0.001 to identify SNPs associated with the traits of interest. For simplicity, only SNPs significant for both survival days and survival status were defined as significant SNPs associated with BCWD resistance.

### QTL Mapping

The half-sib regression analysis module implemented in software GridQTL (Seaton et al., 2006) was used to identify QTL for BCWD resistance and spleen index as described in our previous publication. Based on 10,000 permutations, a significant QTL has to be significant (*P* < 0.05) at both the chromosome-wide level and the experiment-wide level; a suggestive QTL is only significant (*P* < 0.05) at the chromosome-wide level but not at the experiment-wide level. The 95% QTL confidence intervals were estimated using 10,000 bootstraps with re-sampling with software GridQTL (Seaton et al., 2006). The proportion of phenotypic variance explained by the QTL was calculated as *h*_q_^2^= 4(1–MSE_full_/MSE_reduced_) where MSE*_full_* and MSE*_reduced_* are the mean squared error of the full and reduced model, respectively.

## Results

### RAD Genotyping and SNP Linkage Maps

Between 6.52 and 10.01 million quality filtered reads were obtained from each mapping parent, and a total of 7,849 SNPs (Supplementary File) were identified. The number of quality filtered reads per offspring ranged from 0.16 to 5.7 million with an average of 2.34 million. Among the 298 offspring genotyped with RAD-seq, only two fish used to measure spleen index had genotype call rates less than 70%. Thus, these two fish were removed from subsequent data analysis. Based on two-point linkage analysis, 7,595 SNPs were assigned to the 29 chromosomes of rainbow trout. The rest of SNPs were either unlinked with other SNPs or were assigned to small linkage groups with unknown chromosome assignments. A total of 638 SNPs were placed on the female linkage maps with a total map length of 5483 cM and an average SNP spacing of 8.6 cM. Likewise, a total of 438 SNPs were placed on the male linkage maps with a total map length of 2964 cM and an average SNP spacing of 6.8 cM. Due to the limitation of genetic mapping software, we used only dam-specific or sire-specific SNPs to construct female or male linkage maps, respectively. Also, to ensure high confidence in the location and order of the markers on each chromosome, very stringent LOD thresholds were used to construct linkage maps. Thus, only a small portion of SNPs were placed on the linkage maps. Nonetheless, more markers were placed on the linkage maps in this study than microsatellites in our previous QTL mapping studies (Vallejo et al., 2014a,b).

### SNPs Associated with BCWD Resistance and Spleen Size based on GWAS

**FIGURE 1:**
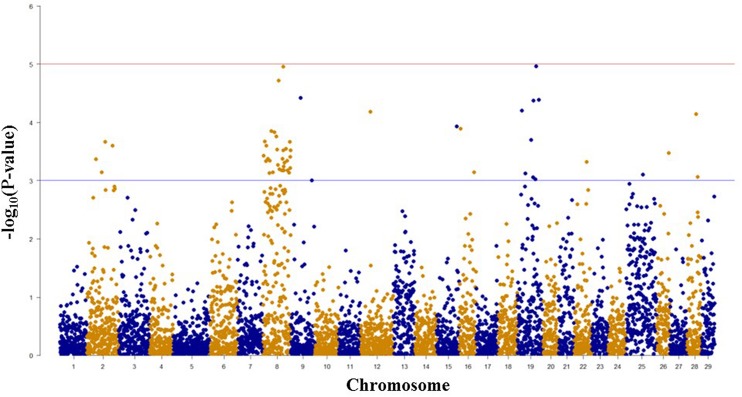
**Results of genome-wide association studies of survival status using GenABEL’s GRAMMAR-Gamma method.** The horizontal lines represent *P* = 1 × 10^-3^ and *P* = 1 × 10^-5^, respectively.

**FIGURE 2:**
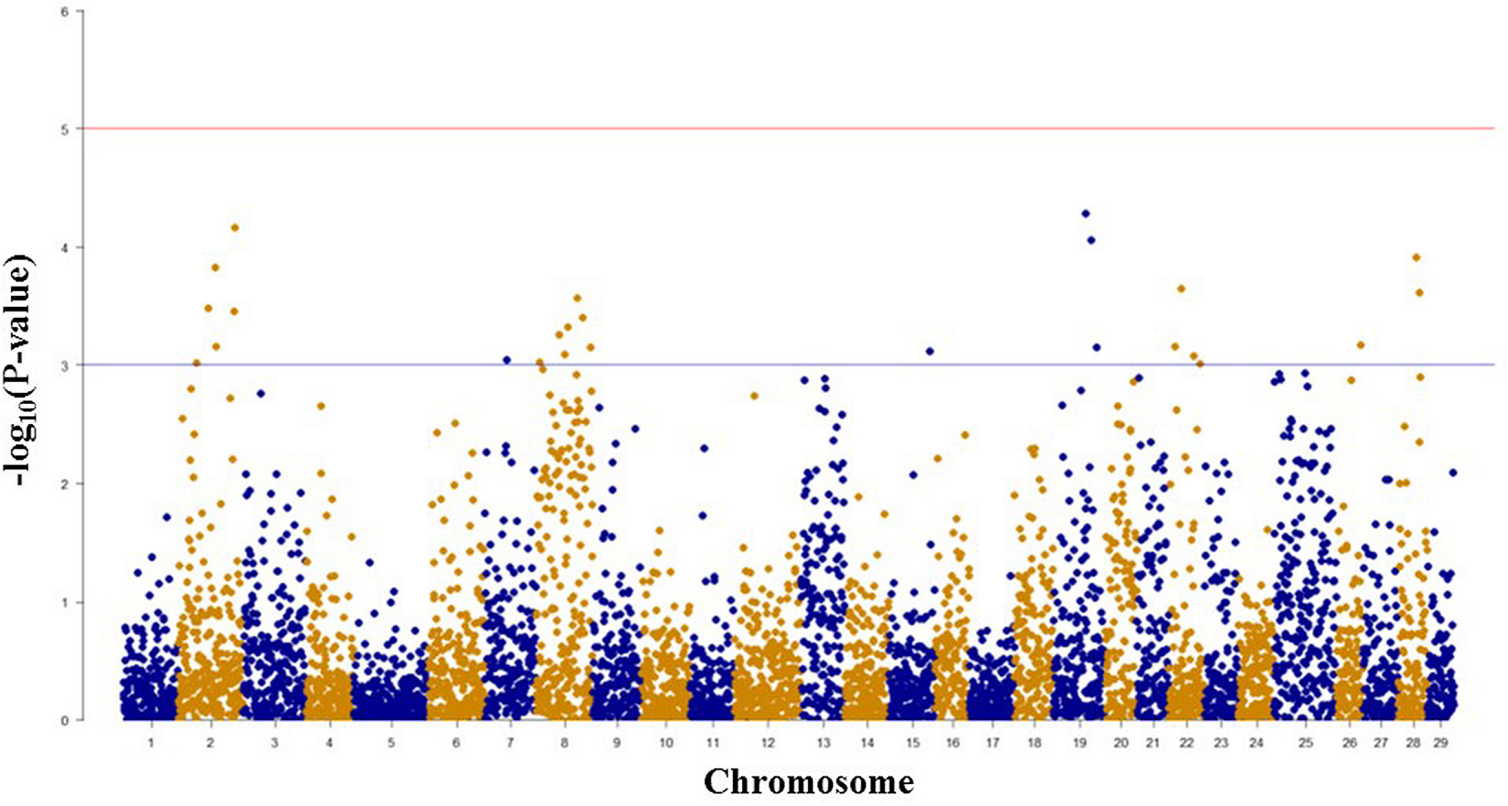
**Results of genome-wide association studies of survival days using GenABEL’s GRAMMAR-Gamma method.** The horizontal lines represent *P* = 1 × 10^-3^ and *P* = 1 × 10^-5^, respectively.

Among 7,595 SNPs assigned to 29 chromosomes of rainbow trout, 5,220 SNPs had genotype call rates over 70% in the combined mapping families 2012473 and 2012474, and were used for GWAS to identify SNPs associated BCWD resistance. Of the 5,220 SNPs analyzed, 18 SNPs associated with BCWD resistance were identified (**Figures 1** and **2**, **Table 1**). As expected, three SNPs on chromosome Omy19 were associated with BCWD resistance. Surprisingly, seven SNPs on chromosome Omy8 were also associated with BCWD resistance. The other eight significant SNPs for BCWD resistance were located on chromosomes Omy2, Omy15, Omy22, Omy26, and Omy28 with only one to three significant SNPs on each chromosome.

**Table 1:** Single nucleotide polymorphism markers associated with bacterial cold water disease resistance based on genome-wide association studies.

				Survival status		Survival days	
SNP	Chromosome	Allele 1	Allele 2	Effect^a^	*P*	Effect^a^	*P*
RTRAD89nt118565	2	T	C	-0.36	2.15E-04	-4.95	1.49E-04
RTRAD89nt095147	2	A	G	-0.34	4.30E-04	-4.29	9.68E-04
RTRAD89nt110567	2	G	T	-0.33	7.24E-04	-4.68	3.31E-04
RTRAD89nt131357	8	C	T	0.33	1.10E-05	3.70	2.72E-04
RTRAD89nt116978	8	G	C	0.40	1.93E-05	4.37	4.77E-04
RTRAD89nt103341	8	T	C	0.45	1.47E-04	5.44	5.53E-04
RTRAD89nt078368	8	A	C	0.42	2.15E-04	5.07	9.48E-04
RTRAD89nt148358	8	C	T	0.36	2.17E-04	4.43	7.16E-04
RTRAD89nt140425	8	A	G	0.44	2.87E-04	5.72	3.98E-04
RTRAD89nt112550	8	G	T	0.42	2.92E-04	5.23	8.18E-04
RTRAD89nt139370	15	G	C	0.38	1.17E-04	4.50	7.63E-04
RTRAD89nt135689	19	C	T	0.43	1.09E-05	5.09	8.76E-05
RTRAD89nt141241	19	G	A	-0.40	4.13E-05	-4.39	7.07E-04
RTRAD89nt125108	19	G	A	0.40	4.21E-05	5.29	5.22E-05
RTRAD89nt127892	22	T	C	-0.23	4.78E-04	-3.03	8.41E-04
RTRAD89nt142090	26	G	A	-0.30	3.36E-04	-3.83	6.82E-04
RTRAD89nt128385	28	G	A	-0.46	7.26E-05	-5.93	1.23E-04
RTRAD89nt137710	28	C	T	-0.26	8.68E-04	-3.77	2.43E-04

*^a^Effect of allele 2 on BCWD resistance phenotypes. Negative value means that allele 1 is associated with BCWD resistance.*

**FIGURE 3:**
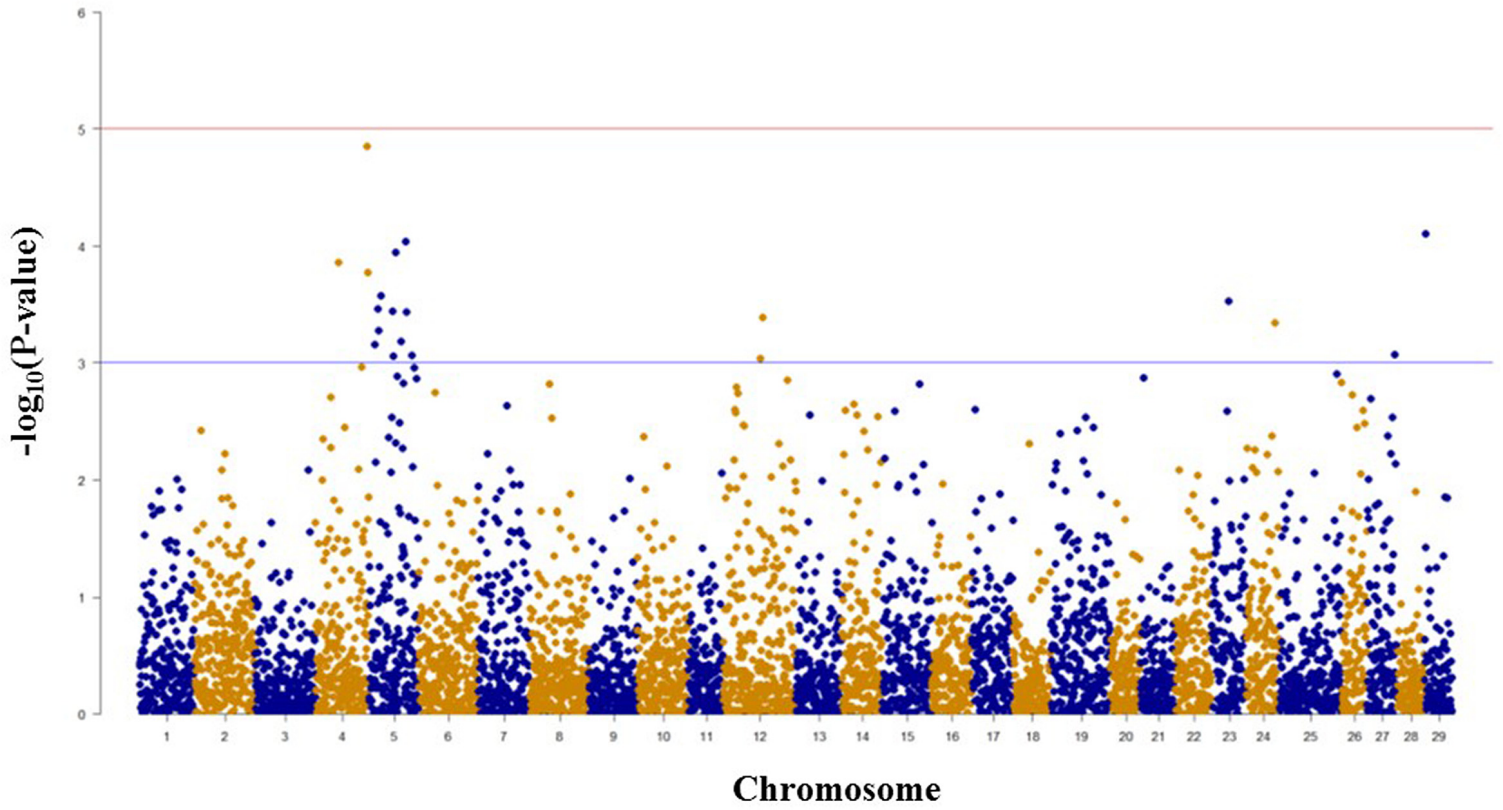
**Results of genome-wide association studies of spleen index using GenABEL’s GRAMMAR-Gamma method.** The horizontal lines represent *P* = 1 × 10^-3^ and *P* = 1 × 10^-5^, respectively.

Among 5,478 SNPs with genotype call rates over 70% in the mapping family 2012474, 20 SNPs associated with spleen index were identified (**Figure 3**, **Table 2**). Eleven of these 20 SNPs were located on chromosome Omy5, and the other nine SNPs were located on chromosomes Omy4, Omy12, Omy23, Omy24, Omy27, and Omy29 (OmySex) with one to three significant SNPs on each chromosome.

**Table 2:** SNPs associated with spleen index based on GWAS.

SNP	Chromosome	Allele 1	Allele 2	Effect^a^	*P*
RTRAD89nt147353	4	G	A	-0.97	1.42E-05
RTRAD89nt107990	4	G	T	-0.79	1.40E-04
RTRAD89nt148293	4	G	A	-0.82	1.69E-04
RTRAD89nt134509	5	A	G	0.85	9.21E-05
RTRAD89nt114301	5	G	T	0.62	1.14E-04
RTRAD89nt091881	5	T	C	0.80	2.69E-04
RTRAD89nt085023	5	C	T	0.78	3.47E-04
RTRAD89nt109036	5	T	G	0.78	3.63E-04
RTRAD89nt137156	5	A	G	-0.79	3.66E-04
RTRAD89nt089304	5	A	G	0.81	5.31E-04
RTRAD89nt127451	5	G	T	0.80	6.62E-04
RTRAD89nt081765	5	C	A	-0.74	6.99E-04
RTRAD89nt142812	5	C	T	0.52	8.70E-04
RTRAD89nt109928	5	T	C	0.74	8.82E-04
RTRAD89nt112119	12	C	A	-0.75	4.09E-04
RTRAD89nt110052	12	A	T	0.52	9.22E-04
RTRAD89nt109171	23	T	C	-0.50	2.99E-04
RTRAD89nt140132	24	G	A	0.81	4.59E-04
RTRAD89nt142053	27	T	C	0.47	8.56E-04
RTRAD89nt072289	29	C	T	0.98	7.88E-05

*^a^Effect of allele 2 on spleen index. Negative value means that allele 1 is associated with higher spleen index.*

### Linkage-based QTL Mapping for BCWD Resistance and Spleen Size

Quantitative trait loci mapping using the half-sib module of GridQTL revealed three significant QTL for BCWD resistance (**Table 3**). These three significant QTL were located on chromosomes Omy8, Omy19, and Omy25, and they explained 72% (STATUS; Sire), 29% (STATUS; Dam), and 34% (DAYS; Sire) of the phenotypic variance, respectively. Consistent with our previous report (Vallejo et al., 2014b), the QTL on Omy19 was derived from the dam 2008132002. The QTL on chromosomes Omy8 and Omy25 were derived from the sires. Suggestive QTL for BCWD resistance were identified on chromosomes Omy2, Omy13, Omy16, Omy20, Omy26, and Omy28 (**Table 3**). Only one significant QTL for spleen index was identified (**Table 4**), which was located on chromosome Omy2. This significant spleen index QTL explained 63% of the spleen index variation. Eight suggestive QTL for spleen index were also identified in this study (**Table 4**) including a suggestive QTL on Omy19 inherited from the sire.

**Table 3:** Quantitative trait loci for BCWD resistance identified by half-sib QTL mapping.

									Flanking markers	
Category	Chromosome	Trait	Position^a^	*F*-value	F_ChromWide_^d^	F_ExperWide_^e^	*h_q_*^2f^	95% C.I.^g^	Left	Right
Dam HS analysis	2	STATUS	20 cM	10.31^b^	8.44	15.3	0.20	7–203	RTRAD89nt135931	RTRAD89nt118565


	16	STATUS	121 cM	8.79^b^	7.75	15.3	0.17	25–157	RTRAD89nt079624	RTRAD89nt086508
	19	STATUS	22 cM	15.41^c^	7.8	15.3	0.29	0–217	RTRAD89nt135689	RTRAD89nt125108
	25	STATUS	96 cM	11.42^b^	7.64	15.3	0.22	0–136	RTRAD89nt088553	RTRAD89nt114044
	28	STATUS	53 cM	7.46^b^	7.4	15.3	0.15	20–134	RTRAD89nt093730	RTRAD89nt148989
	2	DAYS	24 cM	9.96^b^	8.18	15.09	0.19	12–194	RTRAD89nt135931	RTRAD89nt118565
	19	DAYS	24 cM	14.96^b^	8.08	15.09	0.28	0–206	RTRAD89nt135689	RTRAD89nt125108
	20	DAYS	58 cM	7.31^b^	7.09	15.09	0.14	0–147	RTRAD89nt138373	RTRAD89nt137053
Sire HS analysis	8	STATUS	6 cM	21.46^c^	4.17	8.44	0.72	2–45	RTRAD89nt077533	RTRAD89nt148358


	13	STATUS	131 cM	5.11^b^	4.49	8.44	0.20	4–135	RTRAD89nt116690	RTRAD89nt117459
	25	STATUS	64 cM	6.8^b^	4.81	8.44	0.26	0–105	RTRAD89nt085710	RTRAD89nt120059
	26	STATUS	20 cM	5.35^b^	4.15	8.44	0.21	5–33	RTRAD89nt102658	RTRAD89nt096484
	28	STATUS	80 cM	5.76^b^	4.33	8.44	0.22	2–80	RTRAD89nt083376	RTRAD89nt128385
	8	DAYS	16 cM	12.45^c^	4.1	8.56	0.45	0–49	RTRAD89nt148358	RTRAD89nt142845
	12	DAYS	7 cM	4.7^b^	4.31	8.56	0.18	0–77	RTRAD89nt108132	RTRAD89nt076924
	13	DAYS	132 cM	6.34^b^	4.54	8.56	0.24	5–135	RTRAD89nt116690	RTRAD89nt117459
	25	DAYS	65 cM	9^c^	4.75	8.56	0.34	18–115	RTRAD89nt085710	RTRAD89nt120059
	28	DAYS	80 cM	6.25^b^	4.29	8.56	0.24	14–80	RTRAD89nt083376	RTRAD89nt128385

*^a^The peak position of QTL plot; ^b^suggestive QTL; ^c^significant QTL; ^d^F-value threshold for chromosome-wide significance; ^e^F-value threshold for experiment-wide significance; ^f^phenotypic variance explained by each QTL; ^g^95% confidence interval.*

**Table 4:** Quantitative trait loci for spleen index identified by half-sib QTL mapping.

								Flanking markers	
Category	Chromosome	Position^a^	*F*-value	F_ChromWide_^d^	F_ExperWide_^e^	*h_q_*^2f^	95% C.I.^g^	Left	Right
Dam HS analysis	5	3 cM	11.36^b^	7.7	14.64	0.43	3–194	RTRAD89nt108265	RTRAD89nt137156
	7	130 cM	8.54^b^	8.14	14.64	0.33	12–215	RTRAD89nt136795	RTRAD89nt071431
	26	152 cM	8.67^b^	7.39	14.64	0.34	19–152	RTRAD89nt099530	RTRAD89nt148730
	27	22 cM	7.82^b^	7.25	14.64	0.31	14–142	RTRAD89nt113238	RTRAD89nt132122
Sire HS analysis	2	6 cM	17.6^c^	6.59	13.1	0.63	0–36	RTRAD89nt119684	RTRAD89nt149352
	6	22 cM	9.28^b^	5.95	13.1	0.36	0–59	RTRAD89nt089147	RTRAD89nt077857
	17	52 cM	8.79^b^	6.71	13.1	0.34	36–146	RTRAD89nt074950	RTRAD89nt090428
	19	27 cM	8.88^b^	5.63	13.1	0.35	22–62	RTRAD89nt094659	RTRAD89nt149131
	22	96 cM	7.36^b^	6.53	13.1	0.29	21–96	RTRAD89nt138842	RTRAD89nt109237

*^a^The peak position of QTL plot; ^b^suggestive QTL; ^c^significant QTL; ^d^F-value threshold for chromosome-wide significance; ^e^F-value threshold for experiment-wide significance; ^f^phenotypic variance explained by each QTL; ^g^95% confidence interval.*

## Discussion

Previously, we used seven microsatellites to validate the QTL for BCWD resistance and for spleen index on chromosome Omy19 in two rainbow trout half-sib families (Vallejo et al., 2014b). In this study, we conducted a whole-genome scan in the same two half-sib families using RAD sequencing to identify SNPs associated with the same two traits. Based on GWAS, 18 SNPs associated with BCWD resistance and 20 SNPs associated with spleen index were identified. Linkage-based QTL mapping revealed three significant QTL for BCWD resistance and one significant QTL for spleen index.

### Consistency between the Results of GWAS and Linkage-based QTL Mapping

We used both GWAS and linkage-based QTL mapping to identify SNP markers associated with BCWD resistance or spleen index in this study. Overall, the results between these two approaches were consistent, especially between significant BCWD QTL and chromosomes with three or more significant SNPs identified by GWAS. There were some discrepancies between GWAS and linkage-based QTL mapping for regions that contained only one or two significant SNPs (**Tables 1** and **2**), and we suggest caution in interpretation of results from these regions. Based on GWAS, there were three chromosomes Omy2, Omy8, and Omy19 with three or more significant SNPs for BCWD resistance. Consistent with this result, linkage-based QTL mapping revealed significant QTL for BCWD resistance on chromosomes Omy8 and Omy19, and a suggestive QTL for BCWD resistance on chromosome Omy2. However, we did not identify SNPs associated with BCWD resistance on chromosome 25 based on GWAS. Thus, we suggest caution for the BCWD QTL on chromosome Omy25.

The consistency between GWAS and linkage-based QTL mapping for spleen index is not as clear as that for BCWD resistance. This might be because only 96 fish were used for spleen index in this study. The only significant QTL for spleen index identified in this study was located on chromosome Omy2, and RTRAD89nt119684 was near the peak of the QTL plot. This SNP can only be assigned to chromosome Omy2 using the combined genotype data of mapping families 2012473 and 2012474. Thus, it was excluded from GWAS analysis for spleen index in family 2012474. Based on GWAS, two chromosomes Omy4 and Omy5 had three and 11 significant SNPs for spleen index, respectively. Similar to this result, a suggestive QTL for spleen index was identified on chromosome Omy5. The QTL plot for spleen index peaked with an *F*-value 7.38 on chromosome Omy4, which was almost near the threshold *F*-value 7.43 for a suggestive QTL.

The consistency between GWAS and linkage-based QTL mapping is not totally surprising because samples from two mapping families were used for GWAS in this study. Ideally, large number of unrelated animals should be used for GWAS. In reality, samples for GWAS are often collected from family based selective breeding program. In that case, we suggest using higher number of families and fewer progeny per family. The number of SNPs used for GWAS in this study is also not ideal. Recently, a rainbow trout 57K SNP chip has been developed (Palti et al., 2015a), which provides a better tool for GWAS in rainbow trout.

### The Significant BCWD QTL on Chromosome Omy19

Using microsatellites for QTL mapping, a significant BCWD QTL on Omy19 was initially identified in the mapping family 2008132 (Wiens et al., 2013b), and this QTL was validated in a subsequent generation (Vallejo et al., 2014b). As expected, this QTL was validated again using RAD-seq for genotyping in this study. Therefore, this QTL is robust and is a suitable target for fine mapping and identification of positional candidate genes for BCWD resistance.

It is challenging to identify positional candidate genes for the BCWD QTL on chromosome Omy19 using the current draft genome sequences of rainbow trout. Based on GWAS, three SNPs (RTRAD89nt125108, RTRAD89nt135689, and RTRAD89nt141241) on chromosome Omy19 (**Table 1**) were associated with BCWD resistance in this study. Markers RTRAD89nt125108 and RTRAD89nt135689 flank the peak of the QTL plot of chromosome Omy19. We used the sequences of these three SNPs to query the draft sequences of rainbow trout deposited at http://www.animalgenome.org/repository/aquaculture/ (We refer to this website as animalgenome.org from now on). RTRAD89nt125108 was mapped onto four BAC (bacteria artificial chromosome) sequences, two perfect sequence matches for each SNP allele. Likewise, RTRAD89nt135689 was mapped onto two BAC sequences, one perfect sequence match for each allele of the SNP. RTRAD89nt141241 was mapped to three BAC sequences. Each of these three SNPs was not only mapped to multiple BAC sequences but also was located on at least two BAC physical contigs. For example, the two BAC clones having perfect match with the sequence of allele A of RTRAD89nt125108 were located on BAC physical contig 11226 (Palti et al., 2012). One of the two BACs matching perfectly with the sequence of allele G of RTRAD89nt125108 was located on BAC physical contig 11227 (Palti et al., 2012), the other BAC clone could not be assigned to its BAC physical contig because the full name of the BAC clone was unknown (BACs were sequenced in pools and some sequences could not be traced back to its original BAC clone due to technical challenges). The mapping of each SNP allele to a separate BAC physical contig is not surprising because of the well documented homeology between the terminal ends of chromosomes Omy19 and Omy10 in rainbow trout (Phillips et al., 2006; Rexroad et al., 2008). Previously we have shown that the BCWD QTL on Omy19 is near the end of chromosome and that most microsatellites near the QTL are mapped to both chromosomes Omy19 and Omy10 (Wiens et al., 2013b). More research is needed to determine which BAC sequences are likely on chromosome Omy19 and which BAC sequences belong to Omy10. We also used the sequences of the three SNPs to query the scaffold sequences of rainbow trout recently published by Berthelot et al. (2014). Each SNP identified one positive scaffold (**Table 5**). However, all three scaffold sequences were less than 40 kb and were located on unknown chromosomes of rainbow trout. Hence, improvement of the genome sequence for this QTL region is needed to identify positional candidate genes for this QTL.

**Table 5:** Rainbow trout genome scaffolds (Berthelot et al., 2014) matched by SNPs associated with BCWD resistance on chromosomes Omy8 and Omy19.

SNP	Chromosome	Scaffold	Size (bP)
RTRAD89nt131357	8	scaffold_8633	26559
RTRAD89nt078368	8	scaffold_450	800741
RTRAD89nt148358	8	scaffold_262^a^	1102120
RTRAD89nt140425	8	scaffold_7501	31078
RTRAD89nt116978	8	scaffold_6507	36069
RTRAD89nt103341	8	scaffold_45159	3029
RTRAD89nt135689	19	scaffold_14916	12228
RTRAD89nt141241	19	scaffold_6543	35825
RTRAD89nt125108	19	scaffold_6016	39141

*^a^Single nucleotide polymorphism marker RM20090703790 associated with BCWD resistance in mapping family 2009070 (Palti et al., 2015b) was also located on this scaffold. But, these two SNPs are 713 Kb apart on this scaffold.*

### Additional Significant QTL for BCWD Resistance

In addition to the significant BCWD QTL on chromosome Omy19, two significant BCWD QTL derived from the sires were detected in this study. They were located on chromosomes Omy8 and Omy25, respectively. Both sires used in this study were derived from the mapping family 2009044, and a significant BCWD QTL on chromosome Omy8 and a suggestive BCWD QTL on chromosome Omy25 were reported in our previous study using microsatellites for QTL mapping (Vallejo et al., 2014a). The QTL on chromosome Omy8 explained a large proportion of phenotypic variance in this study. However, it is known that variance explained by QTL could be over-estimated in mapping populations. The QTL effects should be further evaluated in breeding populations. Mapping families 2009044 and 2009070 share a pair of grandparents. A significant QTL for BCWD resistance on chromosome Omy8 was also detected in mapping family 2009070 in our previous study (Vallejo et al., 2014a) and this QTL was validated using RAD-seq in a recent study (Palti et al., 2015b). Moreover, among the 12 SNPs associated with BCWD resistance reported by Campbell et al. (2014), SNP R46637 is also located on chromosome Omy8. We do not know whether R46637 is located in the chromosome Omy8 BCWD QTL region reported in this study. Nonetheless, the significant BCWD QTL on chromosome Omy8 deserve more attentions in the future.

Seven SNPs associated with the BCWD QTL on chromosome Omy8 were identified in this study. We used the seven SNP sequences to query the rainbow trout genome sequences deposited at animalgenome.org. Three SNPs were mapped onto three different BAC clone sequences, respectively. SNP RTRAD89nt148358 was mapped onto three BAC sequences. The other three SNPs had no significant match with the BAC sequences. Among the seven SNPs associated with the BCWD QTL on chromosome Omy8, only one SNP had no match with the rainbow trout scaffold sequences published by Berthelot et al. (2014). The other six SNPs were mapped onto six different scaffold sequences with a total length near 2 Mb (**Table 5**). Thus, improved reference genome sequence and additional mapping studies are needed to identify positional candidate genes for this QTL.

### Non-Pleiotropic QTL for BCWD Resistance and Spleen Index on Chromosome Omy19 of Rainbow Trout

Consistent with the association between BCWD resistance and spleen size (Hadidi et al., 2008), both QTL for BCWD resistance and spleen index on chromosome Omy19 were identified in mapping family 2008132 (Wiens et al., 2013b). However, two separately inherited QTL for BCWD resistance and spleen index on chromosome Omy19 were identified in four additional mapping families we genotyped with seven microsatellites (Vallejo et al., 2014b). In this study, we used RAD-seq for genotyping and 7,849 informative SNPs were identified to further resolve inheritance of these two QTL. Based on GWAS, three SNPs on chromosome Omy19 were significantly associated with BCWD resistance, but there was no significant SNP for spleen index on chromosome Omy19. Similarly, half-sib QTL mapping revealed a significant QTL for BCWD resistance on chromosome Omy19 of fish 2008132002, and there was no spleen index QTL on chromosome Omy19 of fish 2008132002. Therefore, we independently validated our microsatellite-based finding that non-pleiotropic QTL control BCWD resistance and spleen index on chromosome Omy19.

The conclusion of non-pleiotropic QTL controlling BCWD resistance and spleen index on chromosome Omy19 is also supported by the identification of a suggestive QTL for spleen index derived from the sire 2009044044 but no corresponding QTL for BCWD resistance on this chromosome. Moreover, our results also indicated that the non-pleiotropic QTL for BCWD resistance and spleen index might also be extended to other chromosomes. Among the 18 SNPs associated with BCWD resistance and 20 SNPs associated with spleen index identified in this study, none of them was significant for both traits. Although some of the QTL for these two traits were located on the same chromosomes in this study, they were derived from different parents. For example, a significant QTL for spleen index derived from the sire 2009044044 was identified on chromosome Omy2, but the suggestive QTL for BCWD resistance was derived from the dam 2008132002.

The non-pleiotropic relationship between BCWD resistance and spleen index is also consistent with our follow-up studies. Recently, Wiens et al. (2015) reported that reduction of spleen size by splenectomy did not alter BCWD resistance in rainbow trout and thus the greater spleen size in some families does not directly influence resistance, rather it is a non-pleiotropic correlation. The lack of a significant genetic correlation between BCWD resistance and spleen index in our odd-year spawning line of rainbow trout in contrast to the even-year spawning line indicates that the association of these two traits across genetic populations is inconsistent and complex (Wiens et al., 2013b). Given the variable and non-pleiotropic association between these two traits, for future studies, we recommend focusing on mapping the QTL for BCWD post-challenge survival instead of spleen index as the most productive direction for understanding the genetic control of BCWD resistance in rainbow trout.

## Conclusion

Previously, we used seven microsatellites to validate the QTL for BCWD resistance and for spleen index on chromosome Omy19 (Vallejo et al., 2014b). In this study we conducted a whole-genome scan in two half-sib rainbow trout mapping families with the aim to identify SNPs associated with BCWD resistance and spleen size using both GWAS and linkage-based QTL mapping approaches. Among 7,849 SNPs identified in this study, 18 SNPs associated with BCWD resistance were identified by GWAS. In addition to the previously identified dam-derived BCWD resistance QTL on chromosome Omy19 (Wiens et al., 2013b; Vallejo et al., 2014b), we identified two new sire-derived BCWD QTL on chromosomes Omy8 and Omy25, respectively. GWAS analysis also revealed 20 SNPs associated with spleen size. Despite the significant increase of genome coverage in this study, none of these 20 SNPs was located on chromosome Omy19. Furthermore, half-sib QTL mapping did not reveal dam-derived QTL for spleen index on chromosome Omy19 either. These results confirm that BCWD resistance and spleen index are not controlled by pleiotropic QTL on chromosome Omy19. Thus, we should focus on QTL for BCWD resistance instead of spleen size in the future. To facilitate marker-assisted selection for BCWD resistance, we are in the process to validate the SNPs reported in this study in the breeding populations of rainbow trout. The SNP markers reported in this study will also facilitate fine mapping to identify positional candidate genes for BCWD resistance in rainbow trout.

## Conflict of Interest Statement

The authors declare that the research was conducted in the absence of any commercial or financial relationships that could be construed as a potential conflict of interest.

## References

[B1] Aulchenko Y. S. Ripke S. Isaacs A. Van Duijn C. M. (2007). GenABEL: an R library for genorne-wide association analysis. *Bioinformatics* 23 1294–1296. 10.1093/bioinformatics/btm108 17384015

[B2] Baird N. A. Etter P. D. Atwood T. S. Currey M. C. Shiver A. L. Lewis Z. A. (2008). Rapid SNP discovery and genetic mapping using sequenced RAD markers. *PLoS ONE* 3:e3376 10.1371/journal.pone.0003376 PMC2557064 18852878

[B3] Berthelot C. Brunet F. Chalopin D. Juanchich A. Bernard M. Noel B. (2014). The rainbow trout genome provides novel insights into evolution after whole-genome duplication in vertebrates. *Nat. Commun.* 5 3657 10.1038/ncomms4657 PMC4071752 24755649

[B4] Campbell N. R. Lapatra S. E. Overturf K. Towner R. Narum S. R. (2014). Association mapping of disease resistance traits in rainbow trout using restriction site associated DNA sequencing. *G3 (Bethesda)* 4 2473–2481. 10.1534/g3.114.014621 25354781 PMC4267942

[B5] Hadidi S. Glenney G. W. Welch T. J. Silverstein J. T. Wiens G. D. (2008). Spleen size predicts resistance of rainbow trout to *Flavobacterium psychrophilum* challenge. *J. Immunol.* 180 4156–4165. 10.4049/jimmunol.180.6.4156 18322227

[B6] Leeds T. D. Silverstein J. T. Weber G. M. Vallejo R. L. Palti Y. Rexroad C. E. (2010). Response to selection for bacterial cold water disease resistance in rainbow trout. *J. Anim. Sci.* 88 1936–1946. 10.2527/jas.2009-2538 20154172

[B7] Liu S. Vallejo R. L. Gao G. Palti Y. Weber G. M. Hernandez A. (2015). Identification of single-nucleotide polymorphism markers associated with cortisol response to crowding in rainbow trout. *Mar. Biotechnol. (NY)* 17 328–337. 10.1007/s10126-015-9621-4 25652693

[B8] Loch T. P. Faisal M. (2015). Emerging flavobacterial infections in fish: a review. *J. Adv. Res.* 6 283–300. 10.1016/j.jare.2014.10.009 26257926 PMC4522593

[B9] Matise T. C. Perlin M. Chakravarti A. (1994). Automated construction of genetic-linkage maps using an expert-system (multimap) - a human genome linkage map. *Nat. Genet.* 6 384–390. 10.1038/ng0494-384 8054979

[B10] Miller M. R. Dunham J. P. Amores A. Cresko W. A. Johnson E. A. (2007). Rapid and cost-effective polymorphism identification and genotyping using restriction site associated DNA (RAD) markers. *Genome Res.* 17 240–248. 10.1101/gr.5681207 17189378 PMC1781356

[B11] Nematollahi A. Decostere A. Pasmans F. Haesebrouck F. (2003). *Flavobacterium psychrophilum* infections in salmonid fish. *J. Fish Dis.* 26 563–574. 10.1046/j.1365-2761.2003.00488.x 14653314

[B12] Palti Y. Gao G. Liu S. Kent M. P. Lien S. Miller M. R. (2015a). The development and characterization of a 57K single nucleotide polymorphism array for rainbow trout. *Mol. Ecol. Resour.* 15 662–672. 10.1111/1755-0998.12337 25294387

[B13] Palti Y. Vallejo R. L. Gao G. Liu S. Hernandez A. G. Rexroad C. E. III. (2015b). Detection and validation of QTL affecting bacterial cold water disease resistance in rainbow trout using restriction-site associated DNA sequencing. *PLoS ONE* 10:e0138435 10.1371/journal.pone.0138435 PMC4574402 26376182

[B14] Palti Y. Gao G. Miller M. R. Vallejo R. L. Wheeler P. A. Quillet E. (2014). A resource of single-nucleotide polymorphisms for rainbow trout generated by restriction-site associated DNA sequencing of doubled haploids. *Mol. Ecol. Resour.* 14 588–596. 10.1111/1755-0998.12204 24251403

[B15] Palti Y. Genet C. Gao G. T. Hu Y. Q. You F. M. Boussaha M. (2012). A second generation integrated map of the rainbow trout (*Oncorhynchus mykiss*) genome: analysis of conserved synteny with model fish genomes. *Mar. Biotechnol.* 14 343–357. 10.1007/s10126-011-9418-z 22101344

[B16] Phillips R. Nichols K. Dekoning J. Morasch M. Keatley K. Rexroad C. (2006). Assignment of rainbow trout linkage groups to specific chromosomes. *Genetics* 174 1661–1670. 10.1534/genetics.105.055269 16951085 PMC1667062

[B17] Rexroad C. E. III Palti Y. Gahr S. A. Vallejo R. L. (2008). A second generation genetic map for rainbow trout (*Oncorhynchus mykiss*). *BMC Genet.* 9:74 10.1186/1471-2156-9-74 PMC2605456 19019240

[B18] Seaton G. Hernandez J. Grunchec J. A. White I. Allen J. De Koning D. J. (2006). “GridQTL: a grid portal for QTL mapping of compute intensive datasets,” in *Proceedings of the 8th World Congress on Genetics Applied to Livestock Production*, Belo Horizonte.

[B19] Starliper C. E. (2011). Bacterial coldwater disease of fishes caused by *Flavobacterium psychrophilum*. *J. Adv. Res.* 2 97–108. 10.1016/j.jare.2010.04.001

[B20] Svishcheva G. R. Axenovich T. I. Belonogova N. M. Van Duijn C. M. Aulchenko Y. S. (2012). Rapid variance components-based method for whole-genome association analysis. *Nat. Genet.* 44 1166–1170. 10.1038/ng.2410 22983301

[B21] Vallejo R. L. Palti Y. Liu S. X. Evenhuis J. P. Gao G. T. Rexroad C. E. (2014a). Detection of QTL in rainbow trout affecting survival when challenged with *Flavobacterium psychrophilum*. *Mar. Biotechnol.* 16 349–360. 10.1007/s10126-013-9553-9 24241385

[B22] Vallejo R. L. Palti Y. Liu S. X. Marancik D. P. Wiens G. D. (2014b). Validation of linked QTL for bacterial cold water disease resistance and spleen size on rainbow trout chromosome Omy19. *Aquaculture* 432 139–143. 10.1016/j.aquaculture.2014.05.003

[B23] Wiens G. D. Lapatra S. E. Welch T. J. Evenhuis J. P. Rexroad C. E. Leeds T. D. (2013a). On-farm performance of rainbow trout (*Oncorhynchus mykiss*) selectively bred for resistance to bacterial cold water disease: effect of rearing environment on survival phenotype. *Aquaculture* 388 128–136. 10.1016/j.aquaculture.2013.01.018

[B24] Wiens G. D. Vallejo R. L. Leeds T. D. Palti Y. Hadidi S. Liu S. (2013b). Assessment of genetic correlation between bacterial cold water disease resistance and spleen index in a domesticated population of rainbow trout: identification of QTL on chromosome Omy19. *PLoS ONE* 8:e75749 10.1371/journal.pone.0075749 PMC3794016 24130739

[B25] Wiens G. D. Marancik D. P. Zwollo P. Kaattari S. L. (2015). Reduction of rainbow trout spleen size by splenectomy does not alter resistance against bacterial cold water disease. *Dev. Comp. Immunol.* 49 31–37. 10.1016/j.dci.2014.11.003 25445908

